# Heterogeneous Genomic Molecular Clocks in Primates

**DOI:** 10.1371/journal.pgen.0020163

**Published:** 2006-10-06

**Authors:** Seong-Ho Kim, Navin Elango, Charles Warden, Eric Vigoda, Soojin V Yi

**Affiliations:** 1 School of Biology, Georgia Institute of Technology, Atlanta, Georgia, United States of America; 2 College of Computing, Georgia Institute of Technology, Atlanta, Georgia, United States of America; University of Chicago, United States of America

## Abstract

Using data from primates, we show that molecular clocks in sites that have been part of a CpG dinucleotide in recent past (CpG sites) and non-CpG sites are of markedly different nature, reflecting differences in their molecular origins. Notably, single nucleotide substitutions at non-CpG sites show clear generation-time dependency, indicating that most of these substitutions occur by errors during DNA replication. On the other hand, substitutions at CpG sites occur relatively constantly over time, as expected from their primary origin due to methylation. Therefore, molecular clocks are heterogeneous even within a genome. Furthermore, we propose that varying frequencies of CpG dinucleotides in different genomic regions may have contributed significantly to conflicting earlier results on rate constancy of mammalian molecular clock. Our conclusion that different regions of genomes follow different molecular clocks should be considered when inferring divergence times using molecular data and in phylogenetic analysis.

## Synopsis

The rate at which mutations accumulate in a genome, referred as a “molecular clock,” is an instrumental tool in molecular evolution and phylogenetics. Different types of mutations occur via distinctive molecular pathways. In particular, while most mutations occur from errors in DNA replication, spontaneous deamination of methylated CpG dinucleotides is another important source of mutation in mammalian genomes. Molecular clock studies typically combined all types of mutations together. In this paper, the authors analyze molecular clocks of replication-origin and methylation-origin mutations separately. By utilizing high-quality sequence data from several primate species and fossil calibration, the authors demonstrate that the two types of mutations follow statistically different molecular clocks. Methylation-origin mutations accumulate relatively constantly over time, while replication-origin mutations scale with generation-times. Therefore, the *genomic* molecular clock, as a whole, is shaped by the molecular origins of mutations that have accumulated over time. The authors' results have direct implications on phylogenetic analyses, estimation of species divergence dates, and studies of the mechanisms and processes of evolution, where molecular clocks are imperative.

## Introduction

Organisms with longer generation-time tend to exhibit slower molecular clock than those with shorter generation-time, an effect known as “generation-time effect” [1–5]. However, the extent (or even the existence) of generation-time effect is of significant debate [3,6,7]. An opposing theory posits that molecular evolution occurs relatively constantly over time: in other words, molecular clocks are time dependent [6,8]. Here we show that molecular evolution follows both generation-time–dependent and time-dependent molecular clocks, depending upon the molecular origins of the mutations considered.

A generation-time–dependent molecular clock implies that the majority of single nucleotide substitutions in germlines arise from errors during DNA replication [3,9]. However, some mutations may occur independently from DNA replication. This is especially pertinent for transitions at CpG dinucleotides (henceforth, CpG substitutions). CpG substitutions are the most frequent single nucleotide substitutions in vertebrate genomes, accounting for more than a quarter of all substitutions between the genomes of human and chimpanzee [10,11]. Naturally, they play critical roles in several key genetic mechanisms and disease [12–16].

CpG dinucleotides are hypermutable because the cytosines in CpG dinucleotides are targets of DNA methylation in vertebrate genomes [17]. Methylated cytosine rapidly mutates to thymine via spontaneous deamination, causing a C to T (G to A in the complementary strand) transition [17,18]. While DNA replication occurs in a specialized stage of the cell cycle, methylation is not confined to replicating DNA: germline cells are methylated early in their development and stay methylated until global demethylation occurs after fertilization [19,20]. Therefore, methylation-origin mutations will accumulate in a rate proportional to the total amount of time germ cells are methylated between generations. In other words, the molecular clock at CpG dinucleotides should be relatively constant over time.

Indeed, statistical inferences using approximately 2 Mbp of sequence data have suggested that CpG substitutions follow relatively constant molecular clock in mammals [21]. In addition, a recent analysis of male mutation bias in humans and chimpanzees have shown that CpG dinucleotides exhibit much lower male mutation bias than other sites [22]. Since male-mutation bias is caused by the more frequent DNA replications in male germlines compared to female germlines [14], the finding that there is lower male mutation bias in CpG dinucleotides is consistent with the idea that CpG substitutions follow a relatively time-dependent molecular clock.

In this paper, we sought to directly compare genomic molecular clocks of CpG dinucleotides and other sites. To achieve this goal, we focused on catarrhines, specifically two hominoid species (human and chimpanzee) and two Old World monkeys (rhesus macaque and baboon). These four species are chosen because they satisfy two criteria. First, because these species are closely related, we can identify sites that have been part of a CpG dinucleotide in recent past (CpG sites) and other sites with high confidence [23]. Second, hominoids and Old World monkeys have markedly differently generation times. According to Gage [24], average generation times in Old World monkeys is 11.4 years, while in chimpanzees and humans, they are 22 and 28 years, respectively. As a consequence of the difference in generation times, evolutionary rates of replication-dependent substitutions are slower in hominoids than in Old World monkeys [2,4,25].

Utilizing genomic data from these species, we demonstrate that indeed CpG substitutions exhibit a relatively time-dependent molecular clock, in contrast to generation-time–dependent genomic molecular clock. Furthermore, we propose that heterogeneous molecular clocks among different genomic regions may have contributed to conflicting earlier results on the degree of generation-time effect in mammals.

## Results/Discussion

### Slower Molecular Evolution of Hominoid Genomes than Old World Monkey Genomes

We first reevaluated the difference in evolutionary rates between hominoids and Old World monkeys. We analyzed approximately 28 Mbp of genomic sequence alignments to compare rates in human (a hominoid) and baboon (an Old World monkey) using a relative rate test [4,26]. Sequence data from marmoset (a New World monkey) were used as an outgroup. We found that rates in humans are on average 28.4% slower than those in baboons in introns and intergenic regions (Table 1, *p* < 0.001), confirming earlier results [2,4,27]. Because data used in this analysis account for approximately 1% of the human genome and from several different chromosomes, we can conclude that the canonical genomic molecular clocks in primates exhibit significant generation-time effect.

**Table 1:**
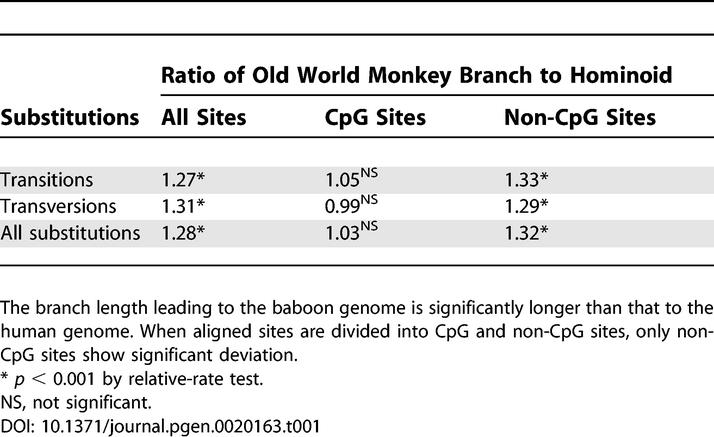
Hominoid-Rate Slowdown Tested Using Genomic Sequence Data from Human, Baboon, and Marmoset

**Figure 1:**
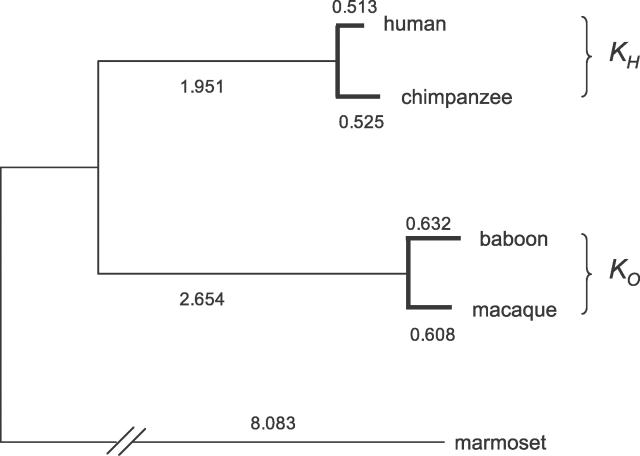
A Neighbor-Joining Tree of Five Primate Species, Generated Using High-Quality Data from the Encode Region ENm001 The numbers of substitutions per 100 sites in each branch, using the two-parameter correction [58], are shown.

We also constructed a five-species phylogeny of human, chimpanzee, baboon, macaque, and marmoset using data for 1.9 Mbp of sequences orthologous to the human chromosome 7 (hg17.chr7: 115404472–117281897; ENCODE region ENm001). High-quality sequence data are available for all five species analyzed in this study. Figure 1 shows a Neighbor-Joining tree [28] of the five species. Focusing on the ancestral hominoid and ancestral Old World monkey branches, the ratio of the number of substitutions in the Old World monkey branch to the hominoid branch is approximately 1.36, similar to the values estimated from the comparison between the human and baboon genomes. These results confirm the “hominoid rate slowdown” theory proposed more than 40 yeasr ago [9,25].

Our next goal was to compare the molecular clocks at CpG and non-CpG sites separately. However, because of the difficulty in correcting for multiple hits, we cannot easily analyze substitutions at CpG sites in this phylogenetic setting. Therefore, we proceeded to use data only in catarrhines, where we can accurately infer rates in CpG and non-CpG sites [12,22,23].

### Different Molecular Clocks of CpG Sites and Non-CpG Sites

**Figure 2:**
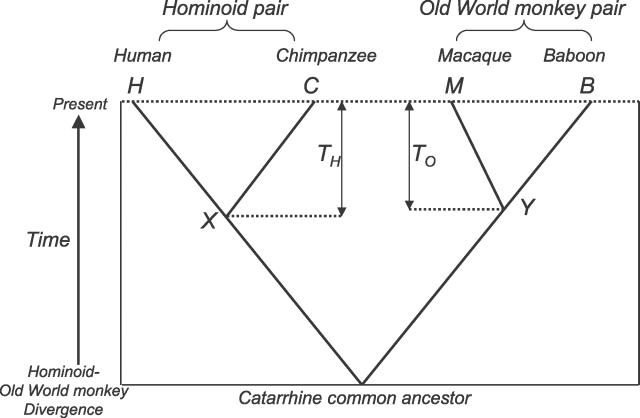
Phylogeny of the Four Taxa Analyzed in This Study *T_O_* denotes the time since the split between the two Old World monkey species, and *T_H_* denotes the time since the split between the two hominoids. Fossil records suggest that *T_O_* and *T_H_* are very close to each other. *X* and *Y* denote the common ancestors of human-chimpanzee and of macaque-baboon, respectively. The genetic divergence between the two hominoid species (*K_H_*) is the sum of *K_HX_* and *K_CX_*. Likewise, *K_O_* is the sum of *K_MY_* and *K_BY_*.

We constructed four-species alignments of two hominoids (human and chimpanzee) and two Old World monkeys (rhesus macaque and baboon) (Figure 2). These species pairs provide a unique opportunity to study time-dependent and generation-time–dependent clocks. Critical to our work, the divergence time between the hominoid pair is similar to that of the Old World monkey pair [27,29,30]. The split between human and chimpanzee is estimated to be 6 to 8 million years ago (Mya), based upon fossil records. In particular, the earliest fossil hominin, *Sahelanthropus tchadensis,* has been dated to late Miocene, at least 7 Mya [30,31]. The split between rhesus macaque and baboon is calibrated by using an estimate for the split between macaques and papionins. The earliest fossil evidence of papionins is dated to be 6 to 8 Mya [27,29]. Therefore, divergence times of the two species within each pair are similar. In other words, *T_O_*/*T_H_* ≈ 1 (Figure 2). In contrast to this similarity of within-pair divergence times, evolutionary rates are known to differ between these two groups: as explained in the introduction and demonstrated above, genomic evolutionary rates in hominoids are slower than rates in Old World monkeys.

We have two contrasting predictions for a time-dependent versus a generation-time–dependent molecular clock. For replication-origin (hence, generation-time–dependent) mutations, the pairwise sequence divergence in the Old World monkey pair (*K_O_* = *K_MY_* + *K_BY_* in Figure 2) should be greater than the pairwise sequence divergence in the hominoid pair (*K_H_* = *K_HX_* + *K_CX_* in Figure 2). On the other hand, a time-dependent molecular clock predicts that *K_O_* is similar to *K_H_*.

**Figure 3:**
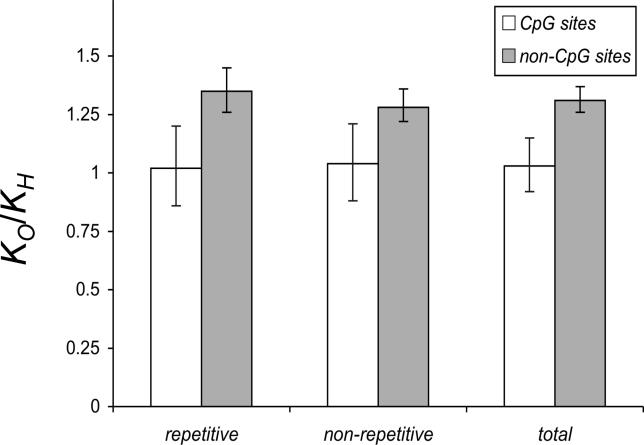
Contrasting Molecular Clocks of Transitions at CpG Sites versus Those at Non-CpG Sites The *y*-axis shows the rate difference in the baboon-macaque pair to that in the human-chimpanzee pair. The Old World monkey pair has accumulated significantly more transitions in non-CpG sites, as expected by the generation time effect. In contrast, transitions at CpG sites, which are primarily of methylation origin, show no difference between the two pairs. Data are shown for all sites, repetitive sites (as identified from the RepeatMasker program [57]), and nonrepetitive sites (after removing repetitive sites). Confidence intervals are generated by bootstrapping 10,000 times.

We examined the molecular clocks in CpG and non-CpG sites separately (see Materials and Methods). To directly compare mutations caused by deamination of methylated cytosines to other transitions occurring during replication, we first analyzed only C-to-T (and G-to-A) transitions. A distinctive pattern emerged: *K_O_*/*K_H_* is 1.03 in CpG sites (95% confidence interval [CI], 0.92 to 1.15), while it is 1.31 in non-CpG sites (95% CI, 1.25 to 1.37). These two types of sites clearly harbor different molecular clocks. Similar trends were discovered when introns and intergenic regions are considered separately, or when repetitive and nonrepetitive sequences are compared separately (Figure 3).

We then considered all single nucleotide substitutions that occurred in CpG and non-CpG sites and found the same pattern. The ratio *K_O_*/*K_H_* in non-CpG sites is 1.18 (95% CI, 1.15 to 1.22). In comparison, in CpG sites, *K_O_*/*K_H_* is 1.00 (95% CI, 0.89 to 1.11). Again, the results are similar when introns and intergenic regions are considered separately, or when repetitive and nonrepetitive sequences are compared separately.

Because human-chimpanzee (hominoid pair) and rhesus macaque-baboon (Old World monkey pair) are extremely closely related, estimates of pairwise sequence divergence are affected by common ancestral polymorphism [32–34]. The common ancestor of the human and chimpanzee is thought to have much larger effective population size than the current human population [35,36]. Rhesus macaque and baboon also harbor comparable levels of genetic diversity to hominoids. For example, Rogers and Kidd [37] reported the nucleotide diversity of Papio hamadryas to be approximately 0.3%. Wall et al. [38] estimated a nucleotide diversity of 0.13% in a noncoding region of rhesus macaques.

Such substantial ancestral polymorphism will effectively reduce the observed rate difference between hominoid and Old World monkey pair: the observed pairwise divergence between rhesus macaque and baboon *(K_O_)* is the sum of ancestral diversity (*π_Y_*, see Figure 2) and the fixed difference between rhesus macaque and baboon (denoted as *P_O_*). Likewise, the pairwise divergence between human and chimpanzee, *K_H_* = *π_X_* + *P_H_*. We are interested in the ratio *P_O_*/*P_H_* while we only have access to *K_O_*/*K_H_*. When comparing distantly related species, the level of ancestral diversity is negligible relative to the fixed difference. However, between closely related species such as human-chimpanzee and macaque-baboon, ancestral diversity is substantial compared to the fixed difference. For example, *π_X_* can be as much as ½ *P_H_* [35]. Therefore, *K_O_*/*K_H_* will underestimate *P_O_*/*P_H_*.

To address this concern, we used the estimates obtained for CpG and non-CpG sites in hominoids [22] to correct for the effect of ancestral polymorphism. After such corrections, *K_O_*/*K_H_* for non-CpG sites is 1.18 to 1.26 (Table 2). In contrast, in CpG sites, *K_O_*/*K_H_* is close to 1.00 even after correcting for the effect of ancestral polymorphism using estimates for CpG sites (Table 2). However, these values should be taken with caution, given the uncertainties associated with ancestral diversity as well as with divergence time estimated from fossil records.

**Table 2:**
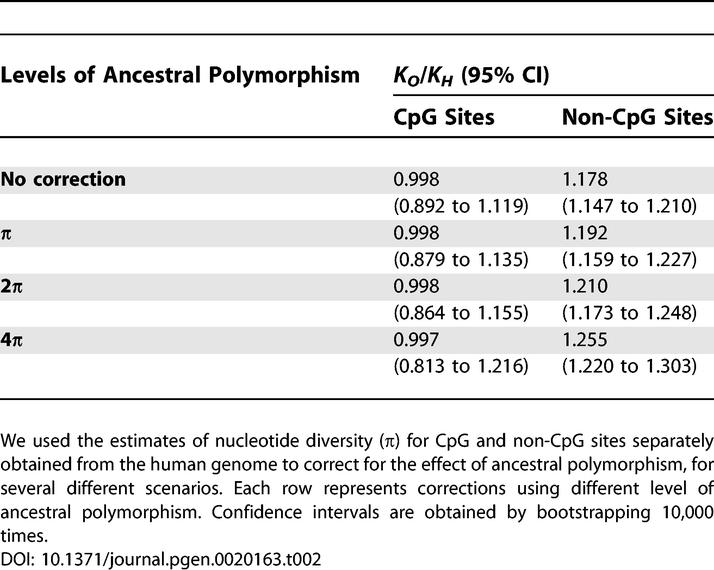
The Ratio of the Pairwise Divergence between Macaque and Baboon *(K_O_)* to the Pairwise Divergence between Human and Chimpanzee *(K_H_),* Using All Substitutions

For completeness, we also analyzed the rate difference for CpG and non-CpG sites using the above three-species alignment (human, baboon, and marmoset). Even though this comparison is less reliable due to the difficulty in correcting for multiple hits (see above), we obtained similar results. We observe that the non-CpG sites (the majority of sites) show substantial rate difference between the human and the baboon genomes. In contrast, CpG sites show little difference in evolutionary rates between hominoid and Old World monkeys (Table 1).

In summary, CpG and non-CpG sites show statistically different molecular clocks in various phylogenetic comparisons, indicating that the difference in two types of molecular clocks is a salient picture of molecular evolution in primate genomes.

### Factors that May Affect *K_O_*/*K_H_* for CpG and Non-CpG Sites

Here we review some of the potential factors that can affect our conclusions. An important assumption in our work is that the divergence time between the hominoid pair is similar to that of the Old World monkey pair. This was mainly based upon fossil records [27,29,30]. However, because fossil records are inherently associated with large variance in dates, let us consider the inference from molecular data.

If we measure the divergence between the Old World monkey pair to that between the hominoid pair in the five species phylogeny shown in Figure 1 (equivalent to *K_O_*/*K_H_* in Figure 2), it is 1.2. This is different from the ratio obtained from the comparison of the ancestral Old World monkey branch to the hominoid branch, which was 1.36. The discrepancy between these two estimates can be explained by at least two mechanisms, which are not mutually exclusive of each other.

First, as mentioned earlier, estimating evolutionary rates between closely related species, such as human-chimpanzee and macaque-baboon, is significantly affected by ancestral polymorphism [32–34]. If we use estimates of the ancestral polymorphism in hominoids [35,36] to correct for the effect of ancestral polymorphism, the ratio of *K_O_*/*K_H_* increases, close to the value estimated from the ancestral branch. For example, if we assume that the average nucleotide diversities of the ancestral Old World monkey and hominoid populations were 0.4%, the corrected ratio of *K_O_*/*K_H_* increases to 1.32.

The second possibility is that the actual time in the Old World monkey pair *(T_O_)* is slightly shorter than the time in the hominoid pair *(T_H_)*. Because fossil records provide only the “minimum” divergence time between lineages, the actual divergence time can differ significantly, and the divergence of human and chimpanzee may have occurred before the divergence of macaque and baboon. Therefore, *K_O_*/*K_H_* will underestimate the true rate difference. According to this possibility, the CpG clock in our data also underestimates the actual rate difference, indicating that some fraction of CpG substitutions follows a generation-time–dependent molecular clock. We believe that this scenario at least partially explains the observed discrepancy, because some substitutions at CpG sites occur during replication. This interpretation is also in accord with the weak but still significant male mutation bias in hominoids [22].

Our study uncovered significant heterogeneity in the degree of generation time effect among different types of single nucleotide substitutions. In particular, when substitutions are divided into transitions and transversions, the latter exhibited less generation-time effect than transitions. In fact, in CpG sites, there were more transversions in the human-chimpanzee pair than in the baboon-macaque pair (58 versus 39). However, the numbers are rather small (since most substitutions at CpG sites are transitions due to methylation), so it is not clear whether this reflects a true underlying pattern. In non-CpG sites, the ratio *K_O_* /*K_H_* estimated from transitions was 1.31, while the ratio from transversions was 1.14 (the overall ratio was 1.18). Whether this discrepancy reflects differences in molecular mechanisms between transitions and transversions is an interesting question and should be pursued further.

### Effect of CpG Dinucleotides on Hominoid Rate Slowdown and Mammalian Molecular Clock

Our findings shed important light on the controversy over mammalian molecular clock. Generation-time effect was clearly demonstrated when closely related species were compared or when noncoding sequences were used [21,27]. However, among relatively distant mammalian species, weak generation-time effect was observed [6,26]. Note that due to sequence availability and alignability, synonymous sites were often used when comparing distantly related species.

We propose that varying proportions of CpG dinucleotides in different data sources can contribute to conflicting conclusions on the nature of genomic molecular clocks. Three observations led to this hypothesis. First, CpG molecular clock runs much faster than clocks at other sites, at least in primates. Assuming that human and chimpanzee diverged 7 Mya [30], we estimate that CpG sites and non-CpG sites undergo single nucleotide substitutions at a rate of 1.03 × 10^−8^ per site per year and 0.68 × 10^−9^ per site per year, respectively, from our data. Second, molecular clocks at CpG sites are relatively constant over time. Third, the proportion of CpG dinucleotides is heterogeneous among different genomic regions [39]. In particular, 4-fold degenerate sites are enriched with CpG sites, over 10% [39], while noncoding regions have less than 3% CpG dinucleotides [22,39]. Hence, molecular clocks in regions with relatively abundant CpG sites (such as 4-fold degenerate sites) may be dominated by the rapid and time-dependent CpG clock, while regions relatively devoid of CpG sites (such as noncoding regions) follow generation-time–dependent molecular clock.

To investigate this prediction, we compared results from different studies in Table 3, focusing on two comparisons: between hominoids and Old World monkeys (hominoid rate slowdown), and between primates and rodents. Note that earlier studies on molecular clock did not consider CpG content as a determinant of molecular clock. Therefore, they did not investigate the effect of CpG content on molecular clock. Because some studies used noncoding regions while others used 4-fold degenerate sites, different studies analyzed different data in relation to CpG content (Table 3). We did not include the results from [6] in this table, because they removed a substantial amount of data that did not pass the “homogeneity test,” and the relationship between this test and CpG dinucleotide content is not clear. For example, they discarded 46% of the data in their human-mouse comparison [6].

**Table 3:**
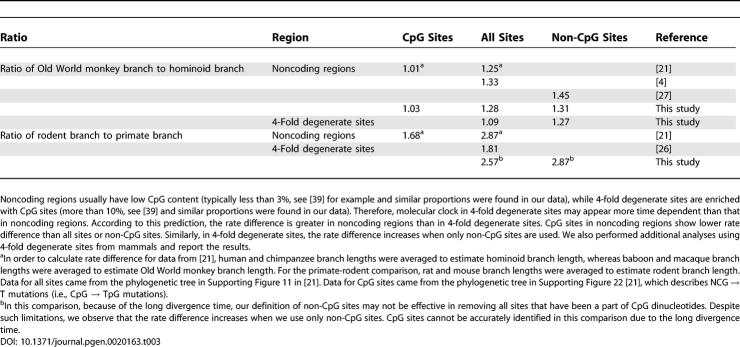
Rate Differences between Lineages from Various Data Sources

We can now compare how the data in Table 3 fit our hypothesis. First, when we compare results from all sites, the rate difference between lineages is greater in noncoding regions than in 4-fold degenerate sites. Moreover, in noncoding regions, the rate difference for CpG sites is lower than for all sites or non-CpG sites. Similarly, in 4-fold degenerate sites, the rate difference in non-CpG sites is higher than in all sites. These trends support our hypothesis.

**Figure 4:**
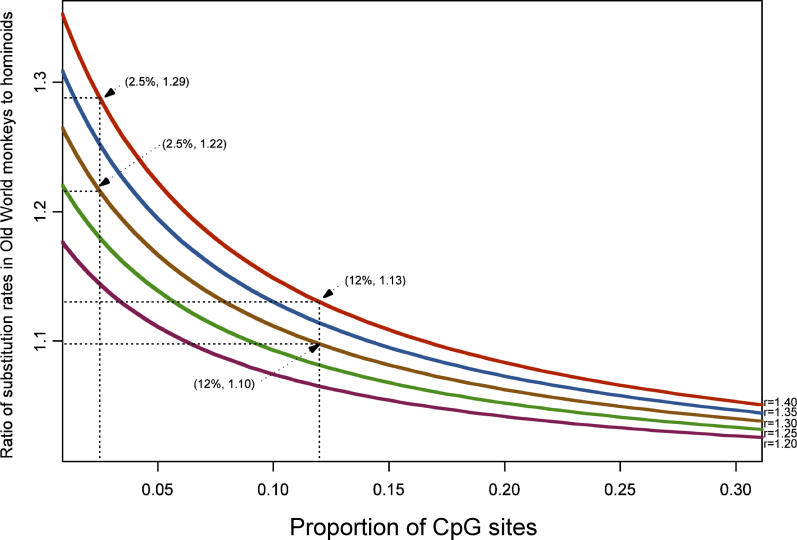
The Proportion of CpG Sites in Data Affects the Degree of Hominoid-Rate Slowdown We considered a simple model in which all sites can be classified into either CpG sites or non-CpG sites and estimated evolutionary rates in hominoids from the human-chimpanzee comparison. The *x*-axis is the proportion of CpG sites in the data. The *y*-axis is the observed degree of hominoid rate slowdown, shown as the ratio of the substitution rate in Old World monkeys to the rate in hominoids, given the “true” ratio (determined by the generation-time effect), depicted as *r*. While regions relatively devoid of CpG sites will reflect the true generation-time effect, the observed ratio approaches 1 as the data include more CpG sites (i.e., the substitution rate in hominoids and Old World monkeys will be similar). Data points for when data consists of 2.5% and 12% CpG sites for *r* = 1.3 and 1.4 are shown for convenience.

Since we have reasonable estimates of CpG and non-CpG rates in primates (see above), we can investigate how well our hypothesis fits the data in detail. The number of substitutions in hominoids since the split from Old World monkeys can be approximated as

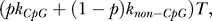
where *p* is the proportion of CpG sites, *k_CpG_* and *k_non-CpG_* represent substitution rates per site per year in CpG sites and non-CpG sites, respectively, and *T* is the time since the split. The observed ratio of Old World monkey branch to hominoid branch can then be expressed as

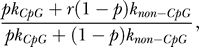
where *r* represents the ratio of the branch lengths determined by the generation-time–dependent molecular clock. Figure 4 shows this ratio as a function of *p*, using the rates inferred from our data. In case when *r* = 1.4, the observed ratios from regions with 12% and 2.5% CpG dinucleotides (analogous to 4-fold degenerate sites and intergenic regions) are 1.12 and 1.29, respectively.

We compared these theoretical expectations to observed values by analyzing rates between hominoids and Old World monkeys in 4-fold degenerate sites, from 41 autosomal genes (Table S2). The proportion of 4-fold degenerate sites that belong to CpG dinucleotides in any of the three species compared in this dataset is 11.0%. This is likely an underestimate of the true proportions of sites that have been part of a CpG dinucleotide, since the divergence time between the three species is rather long. The ratio of the Old World monkey branch to the hominoid branch was 1.09 when all sites were used (Table 3). When we removed CpG-prone sites (sites preceded by C or followed by G, as used in [12,23,40]) from the 4-fold degenerate sites, the aforementioned ratio was increased to 1.27 (Table 3). Recall, when only noncoding sites were used, this ratio was 1.28 (Table 1), which increased to 1.31 when we removed CpG sites. The proportion of sites that belong to CpG dinucleotides in noncoding sites in our data is 2.5%. Therefore, these values are in excellent accord with the above-mentioned model.

It should be noted, however, that the above model ignores other factors that affect regional mutation rate variation, such as GC content and recombination [4,41]. Also, as discussed above, different mutations (such as transitions and transversions) may have different substitution rates between lineages. Hence, partitioning rates into only two categories is likely to be a simplification. Furthermore, identifying sites that have been part of a CpG dinucleotide in the past is a challenging problem [42,43]. Lineage-specific rates are also affected by ancestral generation times and effective population sizes. Further studies are necessary to determine the roles of generation-time–dependent and time-dependent molecular clocks on genome evolution.

Nevertheless, it is clear that the heterogeneity of molecular clocks due to different mutational origins can significantly alter rate differences between taxa. This effect should be taken into account when molecular clocks are used to infer divergence times and to reconstruct phylogenetic history.

## Materials and Methods

### Noncoding data mining and assembly.

Because accurate identification of CpG sites is critical in our analyses, we used two precautions. First, we analyzed sequences between closely related primates only. Earlier studies have shown that within catarrhines (hominoids and Old World monkeys), we can directly derive rates of CpG substitutions using comparative methods. Specifically, we can confidently determine “CpG sites” (sites for which the ancestral state was part of a CpG) and extract rates of CpG substitutions using parsimony [12,22,23]. Moreover, we can also identify sites that have not been a part of CpG dinucleotides (non-CpG sites), to be used as a control for replication-origin substitutions [12,22,23]. Second, we only used high-quality sequence data, because data obtained from whole genome assemblies include errors in sequencing and assembly that can cause erroneous conclusions regarding rate difference between lineages [34,44].

For the human-baboon-marmoset dataset, we obtained approximately 28 Mbp of high-quality data (BAC-based) from the ENCODE project [45].

For the human-chimpanzee-baboon-macaque (HCBM) dataset, we mined high-quality BAC-based sequences from GenBank. The HCBM dataset consists of BAC-based sequence data orthologous to human Chromosome 7 (hg17.chr7:114505472–117281897; Encode region ENm001). This is obtained by aligning NT_086357.2 [46], NT_165329.1 (chimpanzee), NT_086378.3 (baboon), and NT_165339.1 (macaque) sequences.

We assembled additional orthologous alignments among the four species using the following procedure. First, we searched the GenBank database for sequences from baboon *(Papio anubis* or *P. hamadryas),* macaque *(Macaca mulatta),* and chimpanzee *(Pan troglodytes)* BAC clones. We obtained sequence data for 377, 276, and 1,641 BACs from baboon, macaque, and chimpanzee, respectively. Next, we identified orthologous BAC clones among these species, using BLAST [47] and other methods as in [48]. We found 25 baboon BAC clones that had both macaque and chimpanzee orthologs. We then localized orthologous human region for each of these 35 orthologous clones using BLAT [49]. We reconfirmed the orthology between baboon, chimpanzee, and macaque BAC clones by ensuring that the regions where these BAC clones independently map to the human genome overlap with each other. We then removed the BAC clones overlapping with ENm001. Finally, we removed sequences from sex chromosomes. As a result, we obtained 16 genomic regions, shown in Table S1.

### Analysis of 4-fold degenerate sites.

For primate comparison, all sequence data for the primate 4-fold degenerate site comparisons were downloaded from GenBank [50]. Accession numbers for all genes used in primate comparison are available in Table S2. A portion of the homologous genes in this dataset was also identified via the HOVERGEN database [51]. Sequence data for the human-mouse-dog comparison were downloaded from the Ensembl database [52]. Any genes that underwent recent gene duplications or did not meet the stringent minimum length of 445 nucleotides were removed from the dataset. Sequences were aligned using CLUSTALW [53] via a BioPerl package [54]. After alignment of homologous genes, any genes containing lineages with a negative *K_4_* value were removed from the dataset.

For primate-rodent comparison, known genes from human, mouse, and dog were downloaded from Ensembl [52]. To find orthologous sequences, we used the OrthoMCL algorithm [55], which uses an all-to-all BLASTP results to generate a graph of orthologs and paralogs. We used default parameters except for E-value < 10^−10^ to ensure orthology. As a result, we constructed 3,494 orthologous gene trios among the three species. The next steps were performed as described in the primate comparison described above.

### Sequence curation, data annotation, and statistical analyses.

CpG islands were identified using the algorithm by Takai and Jones [56] with the following conditions: GC content greater than 55%, observed/expected CpG contents greater than 0.65, length 200 or greater. Since the majority of CpG islands are hypomethylated and do not reflect substitutions of methylation origin, we removed them from further analysis.

Repetitive elements were annotated using the RepeatMasker program [57]. Noncoding regions are identified as in Elango et al [34].

The two-parameter model [58] was used to correct for multiple hits. We used a relative rate test [4,26] to test for rate difference between hominoid and Old World monkeys using New World monkey species as outgroup (Table S2). To compare rate difference between human and mouse, we used dog as an outgroup.

For classification and rate estimation of CpG sites and non-CpG sites, we used the method in Meunier et al. [12] to identify CpG and non-CpG sites. Specifically, CpG sites are defined as the middle base of the following patterns: XNG/XCG/XCG/XCG, with X denoting any nucleotide except C to avoid overlapping CpGs. N can occur in any of the four sequences. Sites fitting the complementary pattern (CGY/CGY/CGY/CNY, Y not G) are also considered as CpG sites. As a control, sites expected to have never been part of a CpG dinucleotides since the last common ancestor of the four species (“non-CpG sites”) are defined as sites not preceded by C nor followed by G [12,22]. Sites that do not satisfy either classification are defined as “ambiguous sites” and excluded from the analysis. A simulation study has shown that this classifying scheme can accurately identify CpG sites and non-CpG sites in catarrhines [23]. Substitutions are then inferred using unweighted parsimony using only such sites. Confidence intervals for estimated rates are derived from bootstrapping 10,000 times.

## Supporting Information

Table S1 Accession Numbers of Orthologous Baboon, Chimpanzee, and Macaque BACs and Their Locations on the Human Genome (hg 17; NCBI build 35) * This region has a partial overlap with ENm001. The portion that overlapped with ENm001 was removed.
(58 KB DOC)

Table S2 Accession Numbers for Genes Used in Primate Fourfold Degenerate Site Comparison (103 KB DOC)

### Accession Numbers

The National Center for Biotechnology Information (NCBI) (http://www.ncbi.nlm.nih.gov) accession numbers for human, chimpanzee, baboon, macaque, and marmoset are NT_086357.2, NT_165329.1, NT_086378.3, NT_165339.1, and NT_086504.2, respectively.

## Abbreviations

CI: confidence interval
Mya: million years ago
