# Supplementary material for: Heterogeneous Genomic Molecular Clocks in Primates
Source: PLoS Genet. 2006 Oct 6;2(10):e163. doi: 10.1371/journal.pgen.0020163 (PMC1592237; doi:10.1371/journal.pgen.0020163)
Supplement: Supplementary file 1 [file pgen.0020163.st001.doc]

**Table S1.** Accession numbers of orthologous baboon, chimpanzee and macaque BACs and their locations on the human genome (hg 17; NCBI build 35).

| **Baboon BAC** | **Chimp BAC** | **Macaque BAC** | **Human**  **Chromosome** | **Human**  **Start** | **Human**  **End** |
| --- | --- | --- | --- | --- | --- |
| AC149010.2 | AC158371.2 | AC170421.7 | chr14 | 52971380 | 53198115 |
| AC149109.2 | AC155320.2 | AC172115.2 | chr16 | 60842337 | 61130970 |
| AC149627.2 | AC166329.3 | AC169697.1 | chr18 | 23722496 | 23969444 |
| AC183349.1 | AC166329.3 | AC169700.1 | chr18 | 23795853 | 24091531 |
| AC149841.2 | AC157499.2 | AC169801.2 | chr18 | 59700968 | 59948943 |
| AC157860.2 | AC175823.2 | BX842590.1 | chr19 | 59715046 | 59920366 |
| AC165404.2 | AC165373.2 | AC164920.2 | chr2 | 26740092 | 27065987 |
| AC166617.2 | AC169462.2 | AC169132.2 | chr20 | 3486153 | 3745600 |
| AC153752.2 | AC153303.3 | AC153301.2 | chr20 | 43011761 | 43235965 |
| AC155785.2 | AC159464.1 | AC169813.2 | chr4 | 118649444 | 118926297 |
| AC150015.2 | AC159133.2 | AC170761.3 | chr5 | 55785355 | 55990098 |
| AC099743.3 | AC161287.2 | AC171072.2 | chr7 | 89638064 | 89864957 |
| AC157859.2 | AC157483.2 | AC169703.1 | chr7 | 113874528 | 114116788 |
| AC149461.2 | AC159039.2 | AC169819.2 | chr7 | 114268826 | 114509310 |
| AC147705.3* | AC146115.2 | AC151364.3 | chr7 | 115347024 | 115577167 |
| AC149015.2 | AC158591.2 | AC172195.2 | chr8 | 119184014 | 119416122 |
|  |  |  |  |  |  |

* This region has a partial overlap with ENm001. The portion that overlapped with ENm001 was removed.
