# Supplementary material for: Heterogeneous Genomic Molecular Clocks in Primates
Source: PLoS Genet. 2006 Oct 6;2(10):e163. doi: 10.1371/journal.pgen.0020163 (PMC1592237; doi:10.1371/journal.pgen.0020163)
Supplement: Supplementary file 2 [file pgen.0020163.st002.doc]

**Table S2.** Accession numbers for genes used in primate fourfold degenerate site comparison.

| **Gene** | **Homo** | **Macaca** | **NWM** | **NWM Genus** |
| --- | --- | --- | --- | --- |
| ABO | AY268591 | AF052080 | AY091958 | Saguinus |
| ADCYAP1 | S83513 | AY775945 | AY742807 | Saimiri |
| Aipl1 | NM_014336 | AF296411 | AF296415 | Saimiri |
| ASPM | NM_018136 | AY497013 | AY497015 | Saguinus |
| CAMP | NM_004345 | NM_001033509 | DQ471358 | Callithrix |
| CCR5 | NM_000579 | AF005660 | AF452615 | Saimiri |
| CD4 | BT019811 | D63348 | AF452617 | Saimiri |
| CD46 | AY916779 | U87921 | AF025483 | Saimiri |
| CIAS | NM_004895 | AY338198 | AY338203 | Saimiri |
| CSPG3 | AF026547 | AY650346 | AY665242 | Saimiri |
| DARC | NM_002036 | AF311921 | AF311918 | Saimiri |
| FUT1 | NM_000148 | AF080607 | AY219617 | Saimiri |
| FUT2 | NM_000511 | AF136644 | AF136645 | Callithrix |
| Hath1 | BC069594 | AY650376 | AY665222 | Saimiri |
| IL10 | NM_000572 | AB000514 | AF294758 | Saimiri |
| IL16 | NM_172217 | AF017107 | AF017109 | Saimiri |
| IRBP | M22453 | AJ313476 | AF271424 | Saimiri |
| manic fringe | U94352 | AY650338 | AY665226 | Saimiri |
| MC1R | NM_002386 | AY205099 | AY205127 | Saimiri |
| MCPH1 | BC030702 | AY742816 | AY570949 | Saimiri |
| MSX1 | NM_002448 | DQ067471 | DQ067473 | Saimiri |
| NGFB | NM_002506 | AY091928 | AY665218 | Saimiri |
| NTRK3 | BC013693 | AY742818 | AY665246 | Saimiri |
| ODC1 | AY841870 | AY091984 | AY091985 | Saguinus |
| OXTR | NM_000916 | U82440 | AY091952 | Saguinus |
| PHFAH1B1 | AF400434 | AB083321 | AY665240 | Saimiri |
| POMC | NM_000939 | M19658 | AY091995 | Saguinus |
| Prp/PRNP | AF085477 | AB125193 | U08310 | Saimiri |
| PRSS12 | NM_003619 | AY862980 | AY862982 | Saguinus |
| RH30 | NM_016124 | S70343 | AF012429 | Callithrix |
| RNASE1 | NM_002933 | AF449632 | AF449637 | Saimiri |
| SOD2 | AY267901 | AB201468 | AB087281 | Callithrix |
| Sp17 | Z48570 | AF005551 | AF134585 | Callithrix |
| T2R4 | NM_016944 | AY725015 | AB199025 | Callithrix |
| T2R8 | NM_023918 | AY724999 | AB199060 | Callithrix |
| T2R13 | NM_023920 | AY725009 | AB199115 | Callithrix |
| T2R38 | AY724960 | AY725023 | AB199155 | Callithrix |
| T2R40 | NM_176882 | AY725014 | AB199175 | Callithrix |
| TGM4 | NM_003241 | DQ150493 | DQ150498 | Saguinus |
| ZAN | NM_003386 | AY428851 | AY428855 | Saimiri |
| ZP2 | NM_003460 | Y10690 | Y10767 | Callithrix |
